# Dual-scale assessment of motor dysfunction in older adults with locomotor dysfunction and its association with gait and muscle activation

**DOI:** 10.3389/fnagi.2026.1980454

**Published:** 2026-10-05

**Authors:** Wen Liu, Chunxiao Lu, Lifeng Qian

**Affiliations:** 1 Department of Rehabilitation, Jiaxing Hospital of Traditional Chinese Medicine, Jiaxing, Zhejiang, China; 2 School of Rehabilitation Medicine, Shandong University of Traditional Chinese Medicine, Jinan, Shandong, China

**Keywords:** gait analysis, older adults with locomotor dysfunction, rehabilitation assessment of motor function, scale research, surface electromyography

## Abstract

**Objective:**

This study examined the reliability and validity of a combined 25-question geriatric locomotive function scale (GLFS-25) and Locomotion Dysfunction Grade scale (LDG) classification protocol in older adults with locomotor dysfunction, and explored which objective gait and muscle activation parameters were most strongly associated with GLFS-25 scores.

**Methods:**

The GLFS-25 and LDG were used to assess 289 older adults with locomotor dysfunction, of whom 60 underwent gait analysis and surface electromyography (sEMG).

**Results:**

Reliability analysis yielded a Cronbach’s α of 0.702 for the GLFS-25, indicating acceptable but modest internal consistency. Exploratory factor analysis extracted nine factors, accounting for 55.193% of the cumulative variance. Confirmatory factor analysis showed X^2^/df = 1.424, RMSEA = 0.038 (90% CI: 0.000–0.060), and SRMR = 0.055, indicating acceptable fit; however, CFI = 0.821, TLI = 0.801, and IFI = 0.830 were below 0.90, suggesting partial rather than strong construct validity. In exploratory ridge regression, the model showed an in-sample R^2^ of 0.858, which should be interpreted with caution. Step length showed a positive association with the GLFS-25 score, while knee flexion during swing and the ratio of pressure onset time of the middle foot showed negative associations (*P* < 0.05). However, individual coefficients should be interpreted cautiously.

**Conclusion:**

The GLFS-25 demonstrated acceptable but modest reliability and partial construct validity in this population. In exploratory analyses, its scores showed a strong in-sample association with objective gait and sEMG measures. These findings are hypothesis-generating and require external validation in independent cohorts. Objective measures remain complementary to, rather than substitutes for, subjective assessment. Registration: ChiCTR2400087701.

## 1 Introduction

According to existing researches, older adults with locomotor dysfunction are often described as decreased physical resistance and function, deteriorated organ function, but unrestricted daily self-care, which is an intermediate state between healthy and paralyzed elderly individuals (Bouzaher and El Khoudri, 2024; Jiang et al., 2025; Kang et al., 2025; Tadatoshi et al., 2024; Zhao et al., 2025). There is no clear definition and targeted evaluation scale of this population in the academic community, but the understanding of disability is no longer just about the physiological decline of various organ systems. Instead, it comprehensively measures multiple dimensions such as physiological function, illness, mental state, and psychological cognition.

The 25-question Geriatric Locomotive Function Scale (GLFS-25) is a widely used quantitative scale that correlates well with physical function tests, but it does not provide a clear motor dysfunction grade. In contrast, the Locomotion Dysfunction Grade (LDG) scale is a qualitative measure with good acceptance, recommended by the Japanese long-term care insurance system (Seichi et al., 2012); however, its grading is coarse and lacks precision. Iwaya et al. (2017) jointly analyzed the GLFS-25 and LDG scale in a general elderly population and found a significant correlation between them. However, their combined application has not been extended to older adults with locomotor dysfunction, defined here as those who exhibit motor impairment while retaining basic self-care abilities, nor has it been linked to objective gait or muscle activation parameters. Other assessment tools, such as the Short Physical Performance Battery (SPPB), Berg Balance Scale (BBS), and Timed Up and Go (TUG; Feldner et al., 2019; Huang et al., 2022; McManus et al., 2020), have been combined with surface electromyography (sEMG) and gait analysis (Byun et al., 2019; Mazzetta et al., 2019; Tahir et al., 2022; Zago et al., 2018). Our study applies this combined GLFS-25 and LDG classification framework to older adults with locomotor dysfunction and links GLFS-25 scores to instrumented gait and sEMG parameters, thereby providing a quantitative basis for the subjective assessment.

In this study, we employed the GLFS-25 and LDG as a combined classification framework rather than merging them into a single unified instrument. The GLFS-25 supplies the continuous score, and the LDG contributes the clinical grade. This configuration allows cross-validation between patient-reported outcomes and clinician-assigned severity, and enables the translation of continuous scores into interpretable strata for practical use. This study does not aim to validate a merged GLFS-25–LDG instrument; rather, it evaluates the psychometric properties of the GLFS-25 and explores its associations with objective gait and sEMG parameters. We further pose an open question: whether this combined framework may show stronger correlations with objective biomechanical parameters than either scale alone. This comparative advantage is not tested in the present study.

Prior research has examined single-scale associations without integrating biomechanical measures. To address this gap, we evaluated the reliability and validity of the GLFS-25 and performed gait analysis and sEMG on the lower limbs of older adults with locomotor dysfunction. We related these objective metrics to GLFS-25 scores to explore potential indicators of lower-extremity motor dysfunction. Such indicators may provide complementary quantitative information that aids in interpreting subjective scores. However, objective measures are not substitutes for the subjective scale, nor a means to validate it, because the two approaches do not assess the same underlying construct.

## 2 Materials and methods

### 2.1 Reliability and validity test

The GLFS-25 consisted of 25 items and 4 dimensions. A score ≥16 is considered to indicate motor dysfunction, and higher score indicate worse motor function (Seichi et al., 2012). The LDG scale has 6 levels, with levels 1–3 indicating motor dysfunction with the ability to live independently (Iwaya et al., 2017). According to Iwaya et al. (2017), GLFS-25 scores are significantly correlated with LDG grades. Specifically, a GLFS-25 score of 23.34 ± 16.15 was reported for individuals with LDG level 3, and 35.40 ± 19.12 for LDG level 4. Based on these published correspondences and the established GLFS-25 cutoff of ≥16, we defined older adults with locomotor dysfunction as those with a GLFS-25 score between 16 and 35 (inclusive) and an LDG grade of 1–3, indicating motor impairment with preserved basic self-care. This operational definition differs from the broader Locomotive Syndrome concept, which includes individuals with milder symptoms (GLFS-25 ≥ 7) and those requiring heavier care (LDG 4-6).

To reduce heterogeneity, we stratified participants into mild (16–25) and moderate (26–35) subgroups based on the percentile distribution of scores. These strata were used for descriptive purposes only and were not entered into the regression or correlation analyses. Older adults with GLFS-25 scores below 16 were classified as healthy elderly and were excluded from the primary analyses and the gait/sEMG subset. When the GLFS-25 and LDG classifications disagreed, the GLFS-25 score was retained as the continuous measure, and the LDG grade was retained for descriptive and population-definition purposes. No composite score was created.

According to the scoring criteria, this study conducted a questionnaire survey among older adults with locomotor dysfunction selected using sampling methods at the China Rehabilitation Research Center (CRRC). Participants self-administered the GLFS-25, with assistance from trained interviewers when needed. Two rehabilitation physicians independently rated the LDG based on physical examination and functional observation, blinded to the GLFS-25 scores. The inclusion criteria were as follows: (a) aged 60–80 years; (b) a GLFS-25 score between 16 and 35 points (inclusive) and an LDG grade of 1–3, indicating motor impairment with preserved basic self-care (Iwaya et al., 2017); and (c) no contraindications for examination. The exclusion criteria were as follows: (a) severe brain, cardiovascular, lung or kidney disease; (b) a history of lower extremity or spinal fracture within the past 6 months; (c) treatments for acute trauma; and (d) cognitive impairment and refused to cooperate.

All questionnaires were administered in Mandarin Chinese. The translation and cultural adaptation followed established guidelines. Two independent bilingual translators (one with a medical background and one without) performed forward translation of the GLFS-25 and LDG from English into Chinese. A third bilingual expert synthesized the two forward translations into a single version. Two other different independent translators then performed backward translation into English, and the original and back-translated versions were compared by an expert committee (comprising two rehabilitation physicians, one methodologist, and one linguist) to resolve discrepancies. The translated versions were pilot-tested on 30 older adults (excluded from the main study) to confirm clarity, comprehensibility, and cultural appropriateness. Content validity was evaluated by five rehabilitation experts (three physiatrists and two physical therapists). Each expert rated each item on a 4-point relevance scale (1 = not relevant, 2 = somewhat relevant, 3 = quite relevant, 4 = highly relevant). Item-level content validity index (I-CVI) was calculated as the proportion of experts rating the item as 3 or 4. Scale-level content validity index (S-CVI/Ave) was calculated as the average I-CVI across all items. The pre-specified threshold was I-CVI ≥ 0.78 and S-CVI/Ave ≥ 0.90. Disagreements were resolved through consensus discussion among the expert panel. The final Chinese versions are available at Zenodo: https://doi.org/10.5281/zenodo.19888253. Both scales are published tools widely used in academic research. To our knowledge, they are not subject to any explicit copyright restriction that would prohibit non-commercial academic use. We made reasonable efforts to contact the original developers via the email addresses listed in their publications to request formal permission, but received no response as of the date of submission. Full credit has been given to the original developers in this manuscript.

The minimum sample size for confirmatory factor analysis (CFA) should not be less than 200 cases, while the sample size for exploratory factor analysis (EFA) is generally 5–10 times the number of items. The combined GLFS-25 and LDG classification framework includes 25 items, and allowing for 20% invalid questionnaires, the required sample size was 145–270. Participants were recruited at the CRRC between May 2024 and May 2025. A total of 300 questionnaires were distributed. Eleven questionnaires were excluded because of obvious regular response patterns, leaving 289 valid questionnaires (effective response rate 96.33%). Of these 289 participants, 60 were consecutively recruited for gait and sEMG analysis based on equipment availability and willingness to undergo additional instrumentation. No additional clinical criteria were applied. The remaining 229 participants completed the questionnaire but did not undergo gait or sEMG analysis. Missing data were minimal; questionnaire item missingness was <1%, and no imputation was performed.

The reliability of the GLFS-25 was tested using Cronbach’s α coefficient, and its validity was assessed using content validity and construct validity. The suitability of the sample for factor analysis was assessed using the Kaiser-Meyer-Olkin (KMO) test and Bartlett’s test of sphericity. EFA was conducted using principal component analysis with orthogonal (varimax) rotation, and factors were retained based on the eigenvalue-greater-than-one criterion. CFA was subsequently conducted to evaluate the original four-factor theoretical model of the GLFS-25, not the nine-factor EFA solution. The CFA model was estimated using maximum likelihood estimation on the covariance matrix. The GLFS-25 items were treated as continuous variables, consistent with the original validation studies. Model fit was evaluated using X^2^/df, Root Mean Square Error of Approximation (RMSEA) (with 90% confidence interval), Standardized Root Mean Square Residual (SRMR), Comparative Fit Index (CFI), Tucker-Lewis Index (TLI), and Incremental Fit Index (IFI). No *post hoc* model modifications or correlated errors were included.

### 2.2 Gait and sEMG assessment

Sixty of the 289 participants who completed the questionnaire survey underwent gait analysis and sEMG at the CRRC. A MyoMotion three-dimensional motion acquisition and analysis system (Noraxon, USA; sampling frequency, 100 Hz) and a MyoPressure plantar pressure acquisition and analysis system (Noraxon, USA; sampling frequency, 120 Hz) were used for gait detection. A wireless sEMG tester (Noraxon, USA; sampling frequency, 1500 Hz) was used to detect muscle status. MATLAB (MathWorks, Natick, USA) was used to process the signals (Hallal et al., 2013). Walking was performed along a 10-meter straight walkway at the participant’s self-selected comfortable speed, and participants wore their own comfortable footwear. A minimum of three valid trials were recorded, with rest periods of at least 1 min between trials to avoid fatigue. Data from the dominant side were used for analysis. If the participant had a unilateral impairment, the affected side was used.

Before starting the test, the test process and precautions were explained to participants to ensure that they fully understood and cooperated. Then, the skin was cleaned and scrubbed. In accordance with the recommendations of the guidelines of Surface Electromyography for the Non-Invasive Assessment of Muscles (Hermens et al., 2000), sEMG sensors were attached to the rectus femoris (RF), tibialis anterior (TA), gastrocnemius medialis (GM) and gastrocnemius lateralis (GL), with an inter-electrode distance of 20 mm and electrode placement along the muscle belly. Seven inertial measurement units (IMUs) were placed on the sacrum, lateral femur, lateral tibia and dorsal foot (Niswander et al., 2020; Rekant et al., 2022). IMU calibration was performed according to the manufacturer’s protocol before each session. The tightness of the elastic material was adjusted to ensure that it did not fall off during the test (Rantalainen et al., 2020). During the test, participants were required to stand still for 10 s for posture calibration (Oliveira et al., 2022) and then walk back and forth twice to obtain the average test result. All tests were performed by a rehabilitation specialist, and the room was quiet during the test. The safety of participants was ensured.

The study protocol followed the principles of the Declaration of Helsinki. It was approved by the ethics board of the CRRC (No. 2023-083-01) and registered in the Chinese Clinical Trials Registry (No. ChiCTR2400087701). Written informed consent was obtained from all the participants.

### 2.3 Data collection

Gait and sEMG data were automatically imported into MATLAB for processing and analysis (Tang et al., 2022; Watanabe and Narouei, 2021). Based on clinical relevance, we included four types of parameters: (a) gait spatiotemporal parameters, comprising stance phase, swing phase, foot rotation, step length, time, double stance, stride length, step width, velocity, cadence; (b) kinematic parameters, i.e., joint angles of the hip, knee, and ankle; (c) dynamic parameters, including peak pressure at the heel, middle foot, and anterior foot, as well as the ratio of pressure onset time; and (d) muscle activation parameters, specifically the root mean square (RMS) of the RF, TA, GL, and GM.

Full wave rectification, smooth filtering and RMS processing were performed on the original EMG data successively (Bandini et al., 2023; Liu et al., 2022). Raw sEMG signals were band-pass filtered at 10–450 Hz with a 50 Hz notch filter. Artifact rejection was performed, and trials with excessive noise or sensor displacement were excluded. RMS values were calculated for each gait cycle using a 200 ms moving window and normalized to maximal voluntary contraction obtained from three maximum isometric contractions. After normalization processing, distributions of the RMS values in the gait cycle and the RMS peaks of the standing and swinging phases were obtained. MATLAB used the vertical acceleration data to identify heel impact and extracted the time characteristics of gait. The gait period was then analyzed using the heel impact time, and the data points of each gait period were resampled (Rekant et al., 2022). Within-session reliability for gait parameters ranged from 0.81 to 0.94.

### 2.4 Statistical analysis

All the data were processed using SPSS (version 25.0; IBM Corp., Armonk, NY, USA) and AMOS (version 26.0). The reliability and validity of the GLFS-25 were assessed using Cronbach’s α coefficient, content validity, and construct validity. The reliability and validity analyses were performed on the GLFS-25 items only; the LDG is a separate clinical grading and was not included in these psychometric analyses. *P* < 0.05 was considered statistically significant.

All gait and sEMG parameters were defined as candidate predictors before modeling (Table 2). We used the variance inflation factor (VIF) to assess multicollinearity among the independent variables and confirmed that it was present. To stabilize coefficient estimates while retaining all explanatory variables, we adopted ridge regression. All continuous predictors were centered and standardized (z-scores) before entry into the ridge regression model. The ridge penalty parameter (*K* = 0.02) was selected based on the variance inflation factor criterion (VIF ≤ 10) and inspection of the ridge trace plot. Prior to this, we screened all gait and sEMG parameters against the GLFS-25 total score using Spearman correlation (*P* < 0.05), retaining only those with significant univariate associations for the subsequent multivariate model. No correction for multiple comparisons was applied to these Spearman correlation analyses. All *P*-values reported for these correlations are nominal and uncorrected, and the correlation analysis was exploratory. As a sensitivity analysis, we also performed LASSO regression (alpha = 1) on the same dataset, with 10-fold cross-validation to select the optimal penalty parameter. This LASSO analysis served as a sensitivity check rather than an independent validation, because it used the same data. In the final ridge linear regression model, the GLFS-25 total score served as the dependent variable, and the selected gait spatiotemporal, kinematic, dynamic and muscle activation parameters were entered as independent variables. The correlation and regression analyses were exploratory and unadjusted for potential confounders, including age, sex, body mass index, walking speed, pain severity, comorbidity, and assistive-device use.

The relationship between the GLFS-25 total score and LDG grade was assessed using Spearman correlation. Agreement between the GLFS-25 severity strata and LDG grades was assessed using weighted kappa. Inter-rater reliability of the LDG ratings by the two rehabilitation physicians was assessed using the intraclass correlation coefficient (ICC, two-way random, absolute agreement).

## 3 Results

### 3.1 Reliability and validity analysis

Cronbach’s α-value of the GLFS-25 scale was 0.702, indicating acceptable but modest internal consistency. Across the four dimensions, Cronbach’s α-values were: pain (4 items) = 0.70, activities of daily living = 0.60, social functions = 0.69, and mental health = 0.65. The S-CVI/Ave was 0.92, exceeding the pre-specified threshold of 0.90. All items had I-CVI ≥ 0.78. The KMO yielded a value of 0.735, indicating adequate sampling. Bartlett’s test of sphericity showed X^2^ = 908.403 and *P* < 0.001, confirming the appropriateness of factor analysis (Figure 1). EFA was conducted using principal component analysis with orthogonal rotation. It extracted nine factors with eigenvalues >1, accounting for 55.193% of the total variance. The nine-factor structure is empirically derived and exploratory; it does not replace the original four-factor theoretical model.

**FIGURE 1:**
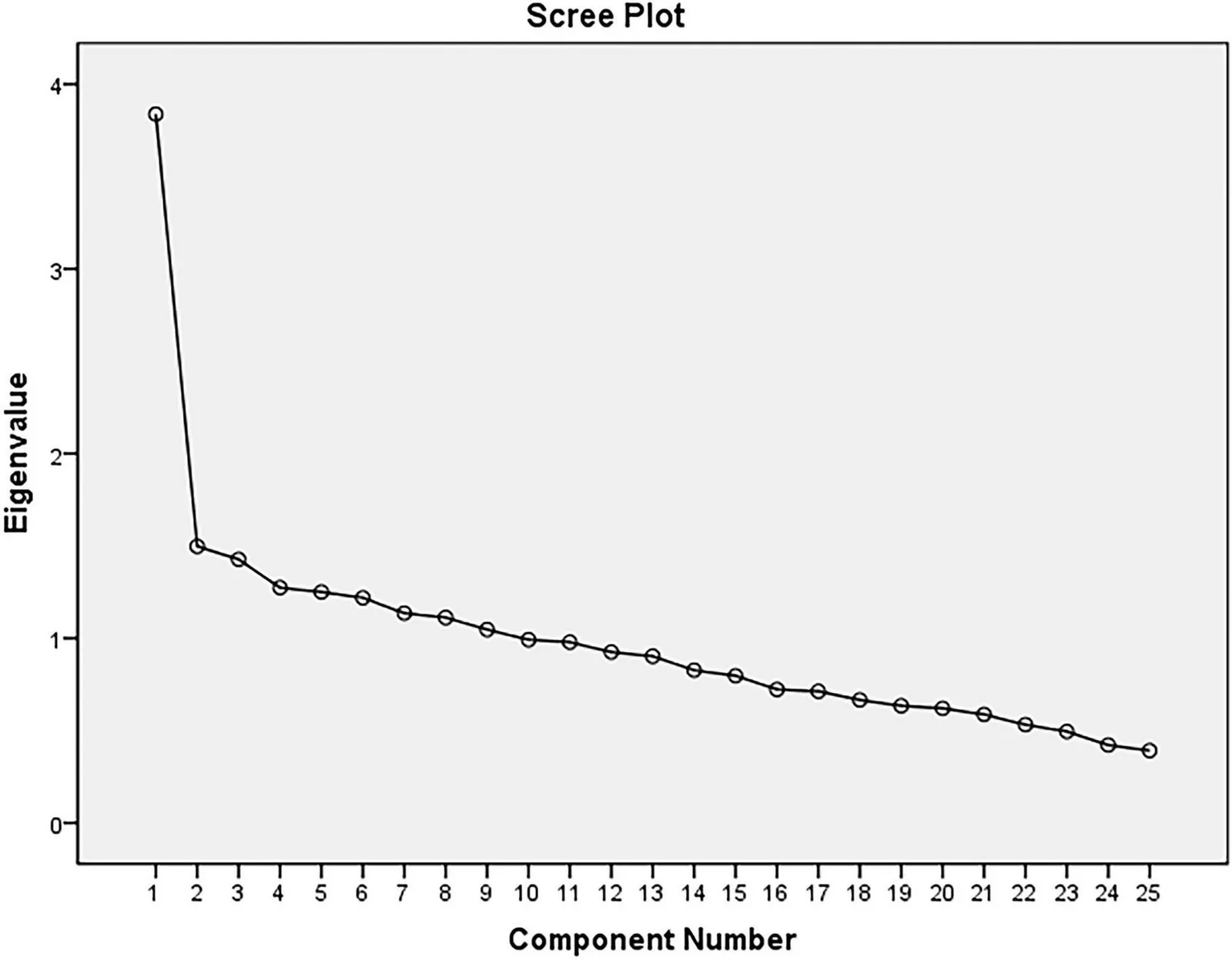
Scree plot.

Confirmatory factor analysis was conducted to evaluate the original four-factor model. The model showed X^2^ = 383, df = 269, X^2^/df = 1.424, RMSEA = 0.038 (90% CI: 0.000–0.060), and SRMR = 0.055, indicating acceptable fit. However, CFI = 0.821, TLI = 0.801, and IFI = 0.830 were below the conventional threshold of 0.90. Standardized factor loadings ranged from 0.40 to 0.81; three items had loadings below 0.50 but were retained because of their theoretical importance and acceptable content validity. All loadings were statistically significant (*P* < 0.01). Overall, the model showed partial rather than strong fit (Figure 2). Full factor loadings, communalities, and cross-loadings are provided in Supplementary Table 1.

**FIGURE 2:**
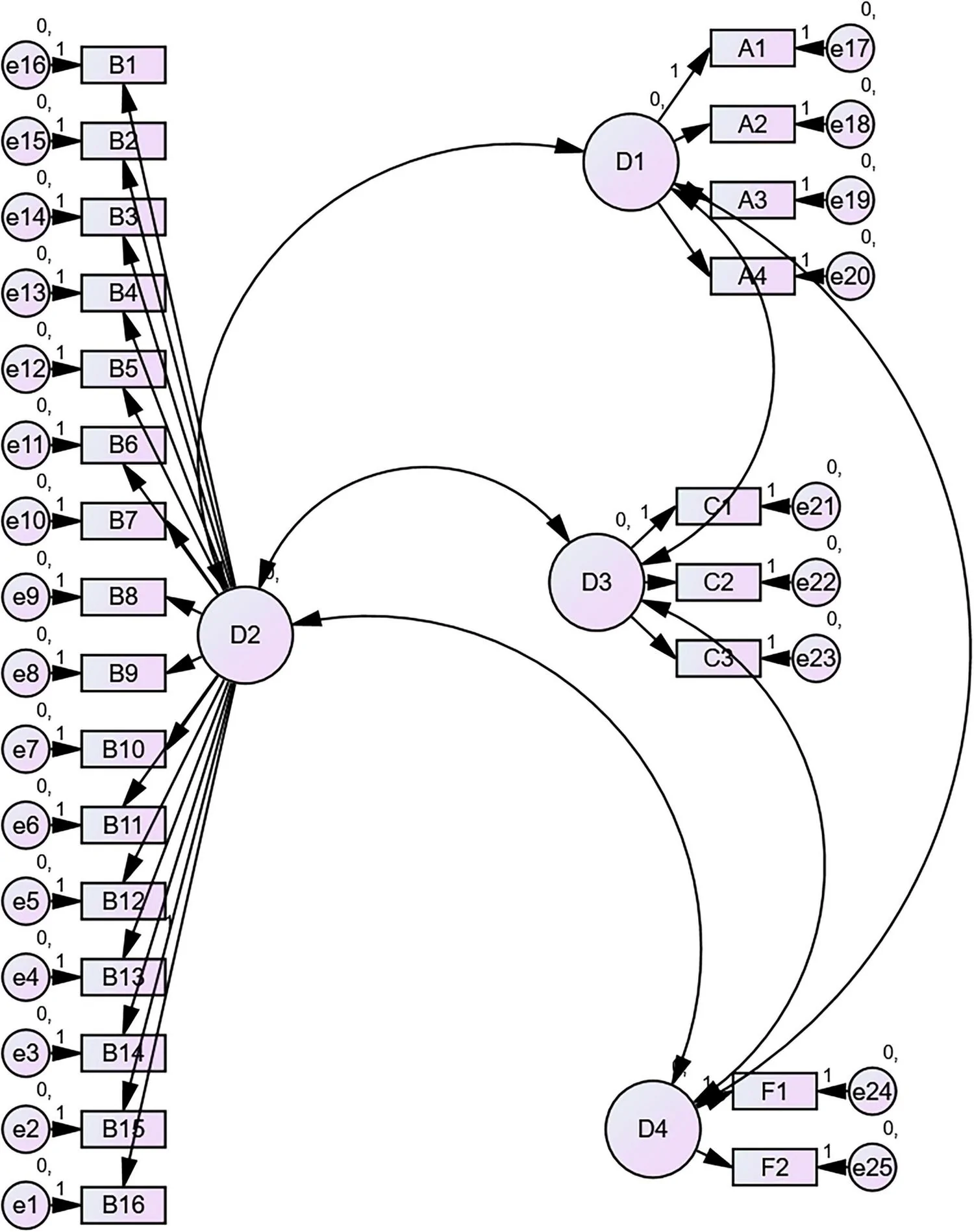
Confirmatory factor analysis path diagram. Standardized coefficients shown; all paths *P* < 0.01. D1 = pain, D2 = activities of daily living, D3 = social functions, D4 = mental health status. RMSEA = 0.038, CFI = 0.821.

Floor and ceiling effects were examined for the GLFS-25 total score. The floor effect was 1.73% (5/289), and the ceiling effect was 3.81% (11/289). These values indicate no notable floor or ceiling effects. The Spearman correlation between the GLFS-25 total score and LDG grade was *r* = 0.72, *P* < 0.001. Weighted kappa for agreement between the GLFS-25 severity strata and LDG grades was 0.52 (95% CI: 0.41–0.63). The intraclass correlation coefficient for inter-rater reliability of LDG ratings between the two rehabilitation physicians was 0.78 (95% CI: 0.68–0.86).

### 3.2 Association analysis

Among the 289 older adults with locomotor dysfunction who completed the questionnaire survey, a subset of 60 underwent additional gait and sEMG analysis. This subset included 29 males and 31 females, with a mean age of 73 (9) years and a mean GLFS-25 score of 23 (14) points. The demographic and clinical characteristics of the full sample and the gait/sEMG subset are summarized in Table 1. There were no significant differences between the gait/sEMG subset and the full sample in age, sex, GLFS-25 score, LDG grade distribution, comorbidity prevalence, or assistive device use (*P* > 0.05; Table 1).

**TABLE 1:** Demographic and clinical characteristics of the full sample and the gait/sEMG subset.

Characteristic	Full sample (*n* = 289)	Gait/sEMG subset (*n* = 60)	*P*
Age, years, median (IQR)#	71.4 (6.8)	73.0 (9.0)	0.612
Sex, male/female	138/151	29/31	0.892
GLFS-25 score, median (IQR)#	24.6 (8.3)	23.0(14.0)	0.945
LDG grade, *n* (%)			0.854
Grade 1	42 (14.5)	8 (13.3)	
Grade 2	113 (39.1)	24 (40.0)	
Grade 3	134 (46.4)	28 (46.7)	
Hypertension, *n* (%)	89 (30.8)	19 (31.7)	0.890
Osteoarthritis, *n* (%)	67 (23.2)	14 (23.3)	0.987
Diabetes, *n* (%)	56 (19.4)	11 (18.3)	0.847
Assistive device use, *n* (%)	34 (11.8)	8 (13.3)	0.732

GLFS-25, 25-question geriatric locomotive function scale; LDG, Locomotion Dysfunction Grade; IQR, interquartile range. #The skewed distribution data are reported as the median and interquartile distances. *P*-values refer to comparisons between the full sample and the gait/sEMG subset.

**TABLE 2:** The association analysis of gait and muscle activation parameters of older adults participants with the GLFS-25 assessment.

Outcomes	Side	95% CI	Spearman	*P*			95% CI	Spearman	*P*
Stance phase (%)	Left	(0.029, 0.778)	0.516	0.049*	Hip flexion	Stance left	(−0.727, 0.242)	−0.335	0.222
	Right	(−0.272, 0.654)	0.217	0.4346		Stance right	(−0.830, −0.136)	−0.564	0.029*
Swing phase (%)	Left	(−0.778, −0.029)	−0.516	0.049*		Swing left	(−0.743, 0.031)	−0.435	0.105
	Right	(−0.654, 0.272)	−0.217	0.436		Swing right	(−0.718, 0.142)	−0.455	0.088
Foot rotation	Left	(−0.318, 0.706)	0.137	0.626	Hip abduction	Stance left	(−0.827, 0.376)	−0.229	0.412
	Right	(−0.230, 0.721)	0.409	0.131		Stance right	(−0.407, 0.742)	0.353	0.197
Step length (cm)	Left	(−0.852, −0.218)	−0.643	0.010**		Swing left	(−0.291, 0.596)	0.143	0.611
	Right	(−0.773, −0.046)	−0.484	0.068		Swing right	(−0.501, 0.498)	−0.026	0.926
Time (s)	Left	(−0.401, 0.750)	0.089	0.753	Hip rotation	Stance left	(−0.481, 0.481)	−0.148	0.598
	Right	(−0.473, 0.611)	0.047	0.869		Stance right	(−0.688, 0.140)	−0.295	0.286
Double stance (%)		(−0.067, 0.775)	0.446	0.095		Swing left	(−0.532, 0.437)	−0.129	0.646
Stride length (cm)		(−0.820, −0.128)	−0.584	0.022*		Swing right	(−0.359, 0.456)	0.074	0.793
Step width (cm)		(−0.089, 0.803)	0.424	0.115	Knee flexion	Stance left	(−0.828, −0.110)	−0.559	0.030*
Velocity (km/h)		(−0.811, 0.040)	−0.491	0.063		Stance right	(−0.909, −0.371)	−0.702	0.004**
Cadence (steeps/min)		(−0.684, 0.409)	−0.150	0.595		Swing left	(−0.787, 0.045)	−0.487	0.066
95% confidence ellipse		(0.138, 0.850)	0.603	0.017*		Swing right	(−0.864, −0.225)	−0.600	0.018*
COP path length		(0.158, 0.831)	0.618	0.014*	Ankle dorsiflexion	Stance left	(−0.785, 0.071)	−0.400	0.140
Average COP velocity		(−0.793, 0.107)	−0.337	0.166		Stance right	(−0.752, 0.083)	−0.448	0.094
peak pressure	The heel left	(−0.638, 0.240)	−0.228	0.414		Swing left	(−0.889, −0.195)	−0.620	0.014*
	The heel right	(−0.781, 0.095)	−0.398	0.142		Swing right	(−0.758, −0.093)	−0.491	0.063
	Middle foot left	(0.239, 0.824)	0.532	0.041*	Ankle inversion	Stance left	(−0.591, 0.400)	0.144	0.608
	Middle foot right	(0.003, 0.756)	0.437	0.103		Stance right	(−0.823, 0.185)	−0.390	0.150
	Anterior foot left	(−0.663, 0.114)	−0.307	0.266		Swing left	(−0.773, 0.413)	−0.255	0.359
	Anterior foot right	(−0.692, −0.001)	−0.397	0.143		Swing right	(−0.684, 0.313)	−0.244	0.380
The ratio of pressure onset time	The heel left	(0.282, 0.812)	0.494	0.061	Ankle abduction	Stance left	(−0.649, 0.485)	−0.197	0.483
	Middle foot left	(−0.319, 0.782)	0.484	0.067		Stance right	(−0.647, 0.332)	−0.155	0.581
	Anterior foot left	(−0.837, 0.011)	−0.500	0.058		Swing left	(−0.844, −0.124)	−0.538	0.039*
	The heel right	(−0.319, 0.782)	0.484	0.067		Swing right	(−0.668, 0.155)	−0.310	0.261
	Middle foot right	(−0.876, −0.471)	−0.690	0.004**					
	Anterior foot right	(−0.873, −0.409)	−0.675	0.006**					
RF	Left RMS (μV)	(−0.542, 0.373)	0.048	0.865	TA	Left RMS (μV)	(0.295, 0.862)	0.645	0.009**
	Left MPF	(−0.520, 0.410)	−0.074	0.793		Left MPF	(0.027, 0.838)	0.459	0.085
	Left MF	(−0.525, 0.409)	−0.076	0.789		Left MF	(0.009, 0.849)	0.454	0.089
	Right RMS (μV)	(−0.528, 0.595)	0.001	0.999		Right RMS (μV)	(0.082, 0.535)	0.328	0.01**
	Right MPF	(−0.390, 0.496)	0.071	0.803		Right MPF	(−0.374, 0.593)	0.186	0.506
	Right MF	(−0.392, 0.531)	0.117	0.678		Right MF	(−0.386, 0.594)	0.141	0.617
GM	Left RMS (μV)	(−0.398, 0.597)	0.093	0.742	GL	Left RMS (μV)	(−0.151, 0.728)	0.332	0.227
	Left MPF	(−0.230, 0.401)	0.243	0.695		Left MPF	(−0.152, 0.744)	0.352	0.198
	Left MF	(−0.350, 0.678)	0.172	0.540		Left MF	(−0.312, 0.369)	−0.127	0.785
	Right RMS (μV)	(−0.708, 0.072)	−0.379	0.163		Right RMS (μV)	(−0.600, 0.395)	−0.094	0.740
	Right MPF	(−0.103, 0.842)	0.458	0.086		Right MPF	(−0.465, 0.615)	0.150	0.595
	Right MF	(−0.139, 0.866)	0.442	0.099		Right MF	(−0.481, 0.544)	0.083	0.767

95% CI, 95% confidence interval; COP, center of pressure; RMS, root mean square; RF, rectus femoris; TA, tibial anterior; GM, gastrocnemius medialis; GL, gastrocnemius lateralis; MPF, average power frequency; MF, median frequency. *Indicates *P* < 0.05, which was considered to indicate statistical significance; **Indicates *P* < 0.01, which was considered to indicate statistical significance. *P*-values are nominal and were not corrected for multiple comparisons. Given the large number of tests, borderline associations (0.04–0.05) should be interpreted with caution and may represent false-positive findings.

The results of Spearman analysis indicated that the spatiotemporal, kinematic, and dynamic parameters and muscle activation parameters of older adults with locomotor dysfunction were correlated with the combined assessment scale (Table 2). Among the gait spatiotemporal parameters, stance phase showed a borderline positive correlation with the GLFS-25 score, while swing phase, step length, and stride length showed borderline negative correlations (*P* < 0.05). These associations should be regarded as exploratory. Among the gait kinematic parameters, the joint angles of the hip, knee, and ankle were negatively correlated with the GLFS-25 score (*P* < 0.05). In the stance phase, hip flexion and knee flexion were significantly correlated with the score; in the swing phase, knee flexion, ankle dorsiflexion, and ankle abduction were significantly correlated with the score. The 95% confidence ellipse, center of pressure (COP) path length, and the GLFS-25 score were positively correlated, while average COP velocity showed no consistent correlation. Among the gait dynamics parameters, the peak pressure at the middle foot was positively correlated with the GLFS-25 score, while the ratio of pressure onset time of middle foot and anterior foot was negatively correlated with the GLFS-25 score (*P* < 0.05).

Among the muscle activation parameters, the RMS peak of the TA showed a positive correlation with the GLFS-25 score (*P* < 0.05). In contrast, the average power frequency (MPF) and median frequency (MF) of the TA, as well as the RMS peak, MPF, and MF of the RF, GM, and GL, showed no significant correlation with the GLFS-25 score (*P* > 0.05).

A total of 42 individual gait and sEMG variables were initially screened against the GLFS-25 total score using Spearman correlation. Of these, 14 variables showed nominal *P* < 0.05 and were entered into the ridge regression model. Through iterative ridge regression, seven predictors were retained in the final model: swing phase, step length, stride length, knee flexion during swing, 95% confidence ellipse, COP path length, and the ratio of pressure onset time of the middle foot. The GLFS-25 total score was used as the dependent variable (Figure 3).

**FIGURE 3:**
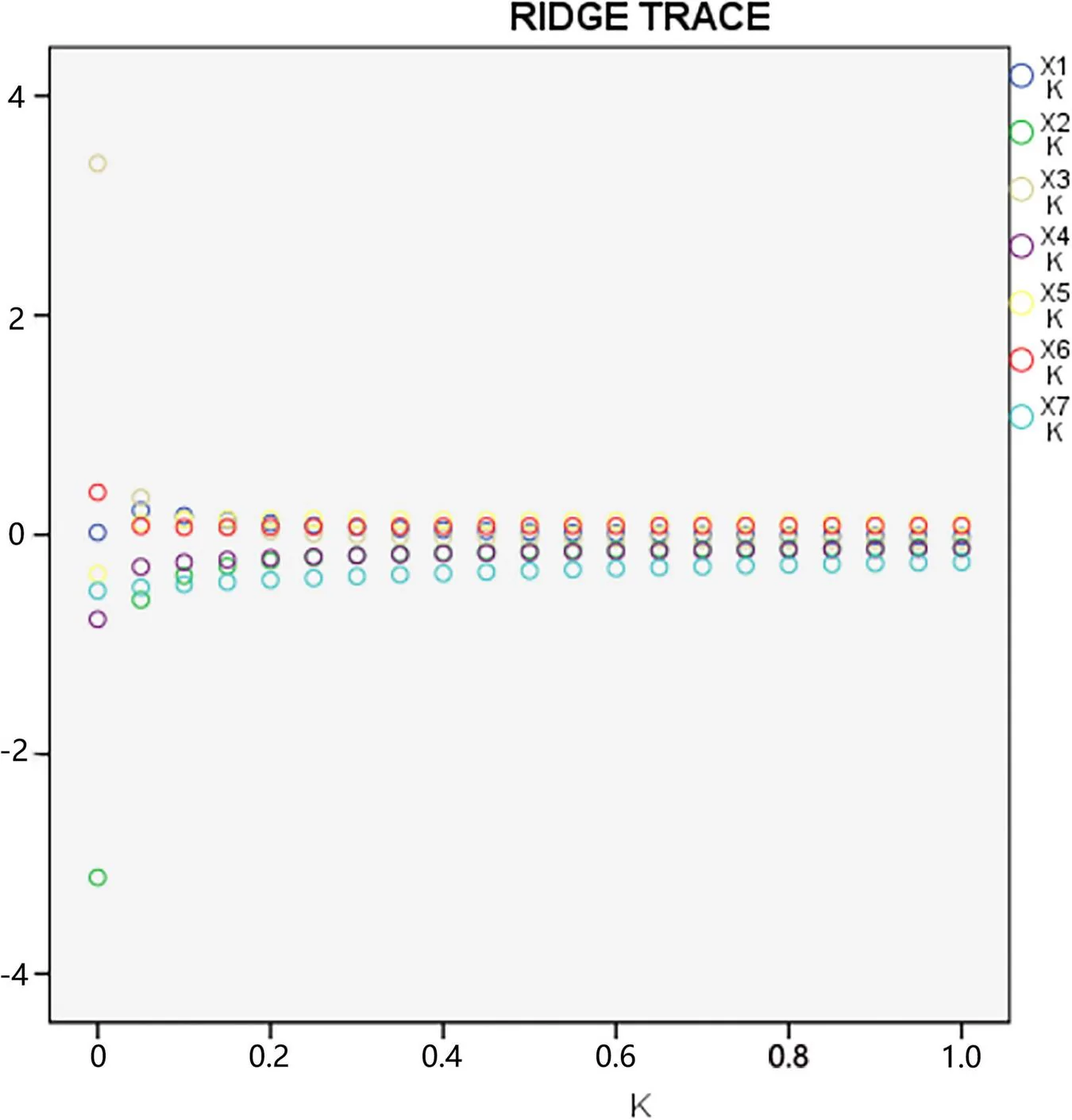
Ridge trace diagram. The horizontal axis represents the ridge penalty parameter (K), and the vertical axis represents the standardized regression coefficient. The vertical dashed line marks the selected penalty value (*K* = 0.02). Predictors: swing phase, step length, stride length, knee flexion swing, 95% confidence ellipse, COP path length, and ratio of pressure onset time of the middle foot.

According to the ridge trace curve, the ridge trace curves for each variable tended to stabilize as K increased. Using the variance inflation factor criterion, the regression coefficient gradually became stable when the variance inflation factor was 7.464 (≤10). Therefore, *K* = 0.02 was adopted to estimate the model parameters. The ridge regression model yielded an R^2^ of 0.858, indicating that the included predictors explained 85.84% of the variance in the GLFS-25 total score. The model *F*-test was significant (*F* = 44.9, *P* = 0.015). The complete output, including non-standardized coefficients, standard errors, standardized coefficients, *t*-values, and *P*-values, is reported in Table 3. The regression equation was: *Y* = 17.463 + 0.439 × swing phase + 0.690 × step length − 0.276 × stride length − 0.000 × 95% confidence ellipse + 0.007 × COP path length − 0.635 × the ratio of the pressure onset time of the middle foot − 0.436 × knee flexion swing. The standardized coefficient for step length was 1.148, which exceeds 1.0 and may indicate multicollinearity or suppressor effects. LASSO regression selected λ = 0.035 (λ.min) and identified the same set of predictors as the ridge model, with similar coefficients: step length = 0.612, knee flexion during swing = −0.398, and ratio of pressure onset time of middle foot = −0.587.

**TABLE 3:** Ridge regression results.

*K* = 0.02	Non-normalized coefficient		Standardization coefficient	t	*P*	R^2^	F	LASSO β
	B	Standard error	Beta					
Independent variables	17.463	14.562	–	1.199	0.269	0.858	*F* = 6.065 *P* = 0.015*	
X1 swing phase	0.439	0.284	0.317	1.546	0.166			
X2 step length	0.690	0.223	1.148	3.093	0.017*			0.612
X3 stride length	−0.276	0.120	−0.891	2.295	0.055			
X4 knee flexion swing	−0.436	0.176	−0.635	−2.471	0.043*			-0.398
X5 95% confidence ellipse	−0.000	0.000	0.251	−0.916	0.390			
X6 COP path length	0.007	0.005	0.569	1.497	0.178			
X7 the ratio of pressure onset time of middle foot	−0.635	0.215	−0.459	−2.955	0.021*			–0.587

Dependent variable: GLFS-25 total score. LASSO coefficients are reported for predictors retained by LASSO. LASSO was performed on the same dataset and serves as a sensitivity check rather than an independent validation.

Among the predictors, swing phase (β = 0.439, *P* = 0.166), stride length (β = -0.276, *P* = 0.055), 95% confidence ellipse (β = -0.000, *P* = 0.390), and COP path length (β = 0.007, *P* = 0.178) were not statistically significant. Step length was positively associated with the GLFS-25 score (β = 0.690, *P* = 0.017; standardized β = 1.148). Knee flexion during swing (β = -0.436, *P* = 0.043; *t* = −2.471) and the ratio of pressure onset time of the middle foot (β = -0.635, *P* = 0.021; *t* = −2.955) were negatively associated with the GLFS-25 score. These coefficients indicate associations, not independent effects, and should be interpreted with caution because of multicollinearity.

## 4 Discussion

We used Cronbach’s α to evaluate the internal consistency of the GLFS-25. The total scale showed acceptable but modest internal consistency, and dimension-level α-values ranged from 0.60 to 0.70. We therefore interpret these values with caution and suggest that future work seek to improve internal consistency. To examine how well the scale structure fitted the theoretical model, we performed confirmatory factor analysis. The model showed acceptable fit for X^2^/df, RMSEA, and SRMR, but CFI, TLI, and IFI were below the conventional threshold of 0.90. Standardized factor loadings ranged from 0.40 to 0.81, with three items below 0.50. Although these three items were retained for theoretical reasons, the overall pattern suggests partial rather than strong construct validity. This is consistent with the dimension-level α-values, which indicate acceptable to modest internal consistency. The original four-factor structure may not fully replicate in this population, and the construct validity findings should be interpreted cautiously. The KMO and Bartlett’s tests supported factorability, but we recognize that these tests do not by themselves establish construct validity. Our evidence for construct validity came from the partial support of CFA and from content validity (CVI = 0.92). The nine-factor structure was empirically derived from EFA using the eigenvalue-greater-than-one criterion. Given the exploratory nature of this analysis, this factor structure awaits confirmation in future theory-driven studies. Test-retest reliability was not assessed in this study. Future studies should evaluate the stability of the GLFS-25 over time in this population.

Several aspects of the factor-analytic strategy warrant caution. First, EFA was conducted using principal component analysis with orthogonal rotation, rather than common factor analysis with oblique rotation. Orthogonal rotation assumes uncorrelated factors, which may not be appropriate for dimensions of physical function that are likely correlated. Second, factors were retained based on the eigenvalue-greater-than-one criterion rather than parallel analysis, which may overestimate the number of factors. Third, EFA and CFA were performed in the same sample, which may overestimate model fit. Ideally, the factor structure should be explored and confirmed in independent samples. Fourth, the GLFS-25 items are ordinal, and we treated them as continuous in the CFA rather than using polychoric correlation matrices or ordinal factor analysis. These limitations should be addressed in future studies.

The combined GLFS-25 and LDG classification protocol is not a single merged scale but a combined assessment. The GLFS-25 supplies the continuous score, whereas the LDG contributes the clinical grade. The factor structure depicted in Figure 2 refers exclusively to the GLFS-25 items. The LDG is a separate clinical grade. Proper interpretation hinges on this distinction. This combined protocol was easy to understand and had an appropriate number of items, allowing participants to complete it within a short time-frame. The effective questionnaire response rate was 96.33%, indicating a high acceptance of the assessment protocol. As the degree of motor impairment increases, the joint stiffness of older adults with locomotor dysfunction intensifies, and their range of motion decreases, resulting in a decline in the body’s balance ability, as well as a decrease in the ability to judge and respond to critical environments, ultimately leading to mobility impairments. If such older adults were not promptly evaluated and given rehabilitation intervention, irreversible motor disability may occur (Shin, 2022). Therefore, rehabilitation assessment and intervention measures related to the motor function of this population are even more important. Using this combined GLFS-25 and LDG classification framework in clinical practice may help rehabilitation workers explore the functional limitations reported by older adults with locomotor dysfunction. However, the present study is cross-sectional and cannot establish that this framework accurately diagnoses motor dysfunction or identifies causal mechanisms.

The GLFS-25 total score was strongly correlated with the LDG grade, consistent with the findings of Iwaya et al. (2017), who reported a significant increase in GLFS-25 scores across LDG grades. The weighted kappa indicated moderate agreement between the GLFS-25 severity strata and LDG grades, suggesting that the two scales capture related but not identical aspects of locomotor dysfunction. The ICC indicated good inter-rater reliability of LDG ratings, supporting the consistency of clinician-assigned grading in this study. These findings support the combined use of the GLFS-25 and LDG for defining the study population.

Previous studies have shown that the combination of gait and sEMG can provide a more comprehensive and objective assessment of lower extremity motor function in older adults and improve assessment accuracy (Commandeur et al., 2024; Liu and Bai, 2024; Schwarz et al., 2023). Therefore, given the satisfactory clinical reliability and validity of the GLFS-25, we used objective gait analysis and sEMG to investigate how scale scores relate to gait characteristics in older adults with locomotor dysfunction. We aimed to generate evidence that could inform clinical classification and quantitative assessment through this subjective-objective combined approach. The biomechanical measures served an exploratory purpose in the association analyses and were not used as a gold-standard validator of the scale. Significant associations were observed for spatiotemporal, kinematic, dynamic, and muscle activation parameters across the study sample (Camerlingo et al., 2024; Elshehabi et al., 2022). Among the sEMG variables, only the RMS peak of the TA showed a positive correlation with the GLFS-25 score, with no other parameters reaching significance. This selective association may suggest that isolated changes in TA activation, rather than generalized lower-limb muscle dysfunction, could be a potential feature of motor impairment in this population. However, because our analysis was exploratory, these findings do not imply that sEMG measures can substitute for the subjective scale.

However, the ridge regression model does not supersede clinical judgment or the combined GLFS-25 and LDG classification framework. Its role is to highlight which biomechanical parameters show the strongest associations with the GLFS-25 scores. The coefficients indicated that step length was positively associated with the GLFS-25 score, whereas knee flexion angle during swing and the ratio of the pressure onset time of the middle foot were negatively associated. In other words, longer step lengths, smaller knee flexion swing angles, and lower pressure onset time of the middle foot corresponded to higher (worse) scores, which may reflect more severe lower-extremity motor dysfunction. That said, correlation is not evidence of interchangeability between the two measurement domains. The GLFS-25 reflects perceived functional limitations, whereas gait and sEMG data capture physical movement patterns. These are distinct but potentially related constructs. We do not advocate using the model equation alone as an independent evaluation tool. Assessment of motor dysfunction in this population should continue to combine subjective reports with clinical examination, while objective parameters serve as adjunct explanatory measures.

The R^2^ value of 0.858 should be interpreted with caution given the modest sample size and the number of predictors, which yielded a ratio of roughly 1–8.6. Ridge regression mitigates over-fitting risk through coefficient shrinkage and collinearity adjustment, yet prudent interpretation remains warranted. We did not compute an adjusted R^2^ or perform cross-validation. In addition, the two-stage strategy used in this study, namely Spearman prescreening followed by ridge regression on selected variables, may introduce selection bias and inflate the explained variance. The correlation analyses in Table 2 involved a large number of tests without correction for multiplicity, so some borderline associations, particularly those with *P*-values around 0.04–0.05, may be false positives. This concern is especially relevant because several borderline variables were subsequently carried forward into the ridge regression model. LASSO regression was performed on the same dataset and therefore does not constitute an independent check, and full-model estimation was not performed.

The standardized coefficient for step length (β = 1.148) exceeded 1.0. Notably, step length showed a negative Spearman correlation with the GLFS-25 total score but a positive standardized ridge coefficient. This sign reversal, together with β > 1.0, indicates substantial multicollinearity or a suppressor effect among the predictors. Conventional *t*-tests, *F*-tests, and *P*-values are not straightforward after data-driven variable selection and ridge penalization. Consequently, the individual regression coefficients should not be interpreted as independent effects of specific gait parameters on the GLFS-25 score. The primary value of the ridge regression model lies in its overall association (R^2^ = 0.858), which is an exploratory in-sample estimate.

The present study does not provide a direct comparison between the GLFS-25 and LDG classification framework and a GLFS-25-only or LDG-only model. Because the ridge regression dependent variable was the GLFS-25 total score and the LDG grade was used only to define the study population, the incremental value of combining the two scales remains untested. The novelty of this study therefore lies in the first application of a combined GLFS-25 plus LDG classification framework in older adults with locomotor dysfunction, and in the exploration of association patterns between GLFS-25 scores and objective gait and sEMG parameters. We do not claim that the combined framework is statistically superior to either scale alone. Future studies should construct a composite dual-scale score or use the LDG as an ordinal outcome to formally test incremental validity.

Human walking is a complex periodic whole-body activity that involves the participation of muscles throughout the body, coordination between the upper and lower extremities, and alternating standing and swinging of the left and right feet. If muscles are damaged, joints and bones need to compensate during walking, which may cause changes in the timing of gait stages and abnormal performance. If there are changes in joints or bones, the proportion of time in each stage would also change accordingly. Numerous studies have underscored the role of enhanced joint mobility of affected hip, knee, and ankle in improving walking ability through rehabilitation therapy (Sittichoke et al., 2019). In our study, older adults with locomotor dysfunction had significantly reduced hip flexion, rotation, knee flexion, ankle inversion, and abduction angles. Early studies have shown that changes in kinematic parameters such as pelvic rotation, hip flexion, and reduced ankle plantar flexion angle were closely related to shorter step length (Judge et al., 1996). The reduction in the range of knee motion in this population was accompanied by a decrease in step length (Hirano et al., 2024a), which may be consistent with the gait training concept proposed by Miyake et al. (2016). By measuring gait spatiotemporal parameters such as velocity and step length, as well as gait kinematic parameters, the association between these indicators and the improvement of gait in this population was further examined, emphasizing their importance in assessing walking ability. Therefore, rehabilitation plans for this population may consider increasing swing time by improving the range of motion of knee flexion and abduction during swing, potentially leading to greater step length and stride length and improved walking ability (Martínez-Montas et al., 2025).

The COP path length, the ratio of the pressure onset time of the middle foot, and 95% confidence ellipse are used as indicators for evaluating posture stability (Jones et al., 2024), reflecting the degree of body sway. The ratio of the pressure onset time of middle foot in older adults with locomotor dysfunction showed a high association with the scale. This may indicate that the gait and posture of this population are poor and that their bodies were prone to large swings and slow down during walking, resulting in lower extremities motor dysfunction and poor walking control ability (Liang et al., 2022). Future studies may consider whether balance, strength, and coordination exercises could improve motor performance in this population; however, the present cross-sectional data cannot establish intervention effects (Hirano et al., 2024b; Rivas-Campo et al., 2023). Age-related and pathological gait changes often coexist in this population. Our sample intentionally excluded severe neurological or orthopedic conditions to isolate motor dysfunction in those with preserved self-care. However, subtle sarcopenia and sensory decline may still confound interpretation. Future studies should stratify by comorbidity burden and include healthy elderly controls to distinguish aging from pathology. Among the sEMG parameters, only the RMS peak of the right TA showed a nominal association with the GLFS-25 total score; no other sEMG parameters reached nominal significance. Because no correction for multiple comparisons was applied, this finding should be regarded as exploratory and requires confirmation in future studies. This may suggest that isolated changes in TA activation, rather than generalized dysfunction across multiple lower-limb muscles, could be a potential feature; however, further studies using co-contraction indices and time-frequency analyses are needed to characterize muscle coordination patterns in this population.

Step velocity is a well-established indicator of walking ability in older adults. In our Spearman analysis, velocity approached but did not reach statistical significance (*P* = 0.063); this may be due to multicollinearity with other gait parameters, and step length, knee flexion during swing, and midfoot pressure timing may offer complementary information. Subjective scale scores and objective biomechanical measures assess fundamentally different constructs. The GLFS-25 and LDG capture perceived functional limitations and clinician judgment, whereas gait and sEMG parameters quantify physical movement patterns. Strong correlations do not imply that these measures are equivalent or interchangeable. Our analysis was exploratory, aiming to identify biomechanical parameters that show the strongest statistical associations with subjective scores. This may provide a mechanistic interpretation of the scale results, but it does not validate the subjective scale against an objective gold standard, nor does it suggest that one type of measure can replace the other. The GLFS-25’s primary validation rests on traditional psychometric methods (reliability, content validity, and construct validity via CFA), which have already been demonstrated.

The GLFS-25 remains a clinical tool whose validation rests primarily on traditional psychometric methods (reliability, content validity, construct validity), which we have already established. These associations may be confounded by age, sex, body mass index, walking speed, pain severity, comorbidity, and assistive-device use. Because our analyses were unadjusted and exploratory, the findings should be interpreted as hypothesis-generating rather than confirmatory.

Several limitations warrant consideration. First, sEMG sensors were prone to displacement, and the sample size was modest, limiting generalizability. No healthy elderly normative database or external criterion measures (e.g., gait speed, TUG) were available. Internal consistency was modest for the total scale and acceptable to modest for the four dimensions; McDonald’s ω, ordinal reliability coefficients, and test-retest reliability were not assessed. Second, the two-stage screening strategy may have introduced selection bias, and no correction for multiple comparisons was applied. Only one sEMG parameter showed a nominal association, and this finding should be regarded as exploratory. LASSO was performed on the same dataset and does not constitute an independent validation. The ridge model was not internally or externally validated; cross-validation was not used to select the penalty parameter, and no adjusted R^2^, bootstrap, or cross-validation procedures were used to assess model optimism. The R^2^ of 0.858 should therefore be regarded as an exploratory in-sample estimate. Third, because the dependent variable was the GLFS-25 total score and the LDG grade was used only to define the study population, no direct comparison with a GLFS-25-only or LDG-only model was performed, and the incremental value of combining the two scales remains untested. The LDG is a separate clinical grading, and its psychometric properties were not evaluated; the factor analyses pertained only to the GLFS-25. Therefore, the present study does not validate a combined GLFS-25 with LDG instrument. Fourth, the factor-analytic strategy had limitations: the exploratory factor analysis used principal component analysis with orthogonal rotation instead of common factor analysis with oblique rotation; factors were retained by eigenvalue >1 rather than parallel analysis; EFA and CFA used the same sample; and the ordinal nature of the items was not considered. Finally, the GLFS-25 score range was restricted to 16–35, cross-cultural invariance was not assessed, no subgroup analyses were performed, and the study lacked an independent clinical reference standard and multicenter recruitment.

## 5 Conclusion

The GLFS-25 demonstrated acceptable but modest reliability and partial construct validity in this population. In exploratory analyses, GLFS-25 scores were associated with several gait parameters, including step length, while most sEMG parameters did not show significant associations. However, because the standardized coefficient for step length exceeded 1.0, the primary finding is the overall association rather than specific coefficient estimates; individual coefficients should therefore not be interpreted as independent effects. Because the LDG was used only to define the study population and was not entered into the regression model, the incremental value of the combined GLFS-25 and LDG classification framework over the GLFS-25 alone remains untested. These findings are hypothesis-generating and require external validation in independent cohorts. The present study does not validate a combined GLFS-25 with LDG instrument, and objective measures should be regarded as complementary to, rather than substitutes for, subjective assessment.

## Data Availability

The original contributions presented in this study are included in this article/Supplementary material, further inquiries can be directed to the corresponding author.
