# Supplementary material for: Dual-scale assessment of motor dysfunction in older adults with locomotor dysfunction and its association with gait and muscle activation
Source: Front Aging Neurosci. 2026 Oct 5;18:1980454. doi: 10.3389/fnagi.2026.1980454 (PMC13677845; doi:10.3389/fnagi.2026.1980454)
Supplement: Supplementary file 1 [file Table_1.docx]

Supplementary Material

# 1 Supplementary Tables

**Supplementary Table 1.** rotated component matrixa

| Component | 1 | 2 | 3 | 4 | 5 | 6 | 7 | 8 | 9 | H2 |
| --- | --- | --- | --- | --- | --- | --- | --- | --- | --- | --- |
| GLFS1 | 0.724 |  |  |  |  |  |  |  |  | 0.747 |
| GLFS2 | 0.694 |  |  |  |  |  |  |  |  | 0.570 |
| GLFS3 | 0.650 |  |  |  |  |  | 0.400 |  |  | 0.463 |
| GLFS4 | 0.502 |  | 0.311 |  |  |  |  |  |  | 0.545 |
| GLFS5 | 0.455 |  |  |  | 0.436 |  |  |  |  | 0.416 |
| GLFS6 | 0.429 | 0.328 |  |  |  |  |  |  |  | 0.574 |
| GLFS7 |  | 0.720 |  |  |  |  |  |  |  | 0.516 |
| GLFS8 |  | 0.609 |  |  |  |  |  |  |  | 0.568 |
| GLFS9 |  | 0.566 |  |  |  |  |  |  |  | 0.552 |
| GLFS10 |  |  | 0.667 |  |  |  |  |  |  | 0.584 |
| GLFS11 |  |  | 0.556 |  |  |  |  |  |  | 0.573 |
| GLFS12 | 0.331 |  | 0.452 |  |  |  |  |  | 0.375 | 0.560 |
| GLFS13 |  |  | -0.349 |  | 0.344 |  |  |  |  | 0.411 |
| GLFS14 |  |  |  | 0.708 |  |  |  |  |  | 0.531 |
| GLFS15 |  |  | 0.424 | 0.495 |  |  |  |  |  | 0.573 |
| GLFS16 |  |  |  |  | 0.725 |  |  |  |  | 0.486 |
| GLFS17 |  |  |  |  | 0.425 |  |  | -0.400 |  | 0.591 |
| GLFS18 |  |  |  |  |  | 0.804 |  |  |  | 0.441 |
| GLFS19 |  |  |  |  |  | 0.739 |  |  |  | 0.656 |
| GLFS20 |  |  |  |  |  |  | 0.716 |  |  | 0.640 |
| GLFS21 |  |  |  | 0.409 |  |  | -0.488 |  |  | 0.522 |
| GLFS22 |  |  |  |  |  |  |  | 0.718 |  | 0.611 |
| GLFS23 |  |  |  | 0.368 |  |  |  | 0.389 |  | 0.477 |
| GLFS24 |  |  |  |  |  |  |  |  | 0.684 | 0.596 |
| GLFS25 | 0.325 |  |  | -0.301 |  |  |  |  | 0.489 | 0.595 |
| Eigenvalue (Total) | 3.838 | 1.497 | 1.427 | 1.273 | 1.250 | 1.219 | 1.136 | 1.112 | 1.047 |  |
| % of Variance | 15.351 | 5.989 | 5.707 | 5.093 | 5.000 | 4.876 | 4.542 | 4.447 | 4.188 |  |
| Cumulative % | 15.351 | 21.340 | 27.048 | 32.141 | 37.141 | 42.017 | 46.559 | 51.006 | 55.194 |  |

Note: rotated component matrix from exploratory factor analysis. Loadings below 0.30 are suppressed. h² = communality. Items with cross-loadings ≥ 0.30 on more than one factor are shown. Eigenvalues and variance explained are provided at the bottom of the table.
